# Leftward Deviation and Asymmetric Speed of Egocentric Judgment between Left and Right Visual Fields

**DOI:** 10.3389/fnins.2017.00364

**Published:** 2017-06-30

**Authors:** Ying Zhou, Bing Li, Gang Wang, Mingsha Zhang, Yujun Pan

**Affiliations:** ^1^Department of Neurology, the First Clinical College of Harbin Medical UniversityHarbin, China; ^2^State Key Laboratory of Cognitive Neuroscience and Learning, Beijing Normal UniversityBeijing, China; ^3^Institute of Neuroscience, Shanghai Institutes for Biological Sciences, Chinese Academy of SciencesShanghai, China

**Keywords:** egocentric reference frame, manual reaction time, subjective straight ahead, eye position, asymmetric perceptions

## Abstract

The egocentric reference frame is essential for body orientation and spatial localization of external objects. Recent neuroimaging and lesion studies have revealed that the right hemisphere of humans may play a more dominant role in processing egocentric information than the left hemisphere. However, previous studies of egocentric discrimination mainly focused on assessing the accuracy of egocentric judgment, leaving its timing unexplored. In addition, most previous studies never monitored the subjects' eye position during the experiments, so the influence of eye position on egocentric judgment could not be excluded. In the present study, we systematically assessed the processing of egocentric information in healthy human subjects by measuring the location of their visual subjective straight ahead (SSA) and their manual reaction time (RT) during fixation (monitored by eye tracker). In an egocentric discrimination task, subjects were required to judge the position of a visual cue relative to the subjective mid-sagittal plane and respond as quickly as possible. We found that the SSA of all subjects deviated to the left side of the body mid-sagittal plane. In addition, all subjects but one showed the longest RT at the location closest to the SSA; and in population, the RTs in the left visual field (VF) were longer than that in the right VF. These results might be due to the right hemisphere's dominant role in processing egocentric information, and its more prominent representation of the ipsilateral VF than that of the left hemisphere.

## Introduction

Even though the external objects can be represented in multiple reference frames (Goodale and Milner, 1992; Olson, 2003; Burgess, 2006; Milner and Goodale, 2008; Land, 2012; Boccia et al., 2014), the egocentric reference frame is the most fundamental one (Filimon, 2015). The egocentric representation of space is elaborated by the integration of visual, auditory, proprioceptive and vestibular information relative to the eye, head, and torso position of the observer (Andersen et al., 1997). Recent functional magnetic resonance imaging studies have found that the right hemisphere has more activity than the left hemisphere when healthy right-handed subjects perform egocentric discrimination tasks (Galati et al., 2000, 2001; Neggers et al., 2006; Chen et al., 2012; Saj et al., 2014). The egocentric judgment is frequently tested by measuring the location of subjective straight ahead (SSA), which subjectively separates the egocentric space into left and right halves. Patients with right hemisphere damage more frequently show egocentric neglect of the contralesional hemispace (Beis et al., 2004; Ringman et al., 2004; Becker and Karnath, 2007; Kleinman et al., 2007), as well as an ipsilesional deviation of the SSA (Karnath, 1994; Farne et al., 1998; Ferber and Karnath, 1999; Schindler and Kerkhoff, 2004; Richard et al., 2004a,b, 2005; Saj et al., 2006; Rousseaux et al., 2013). These findings reveal that the processing of egocentric information is asymmetrically distributed between the two hemispheres, with the right hemisphere playing a more dominant role. However, studies of SSA in healthy subjects showed controversial results (Jeannerod and Biguer, 1989; Karnath et al., 1994, 2002; Chokron and Imbert, 1995; McCourt et al., 1997; Vallar et al., 1999; Richard et al., 2004a,b; Saj et al., 2006, 2013; Sumitani et al., 2007; Reinersmann et al., 2012; Rousseaux et al., 2013).

The inconsistent results of SSA in healthy subjects among previous studies might be caused by the employment of different experimental tasks. Two types of tasks were mainly used to measure the location of SSA: the proprioceptive straight ahead pointing task (Heilman et al., 1983) and the visual straight ahead task (Bridgeman and Graziano, 1989). In the former task, subjects pointed to the subjective mid-sagittal plane. In the latter task, subjects either pressed a button or orally reported when a moving visual stimulus reached the subjective mid-sagittal plane (Karnath et al., 1994; Vallar et al., 1999), or adjusted the position of a visual target to the subjective mid-sagittal plane (Bridgeman and Graziano, 1989; Richard et al., 2004b; Saj et al., 2006). In the former task, since the location of SSA was influenced by the pointing hand (left versus right) and the starting position of the hand (Jeannerod and Biguer, 1989; Chokron and Imbert, 1995; McCourt et al., 1997), the results were diverse among the studies. On the other hand, in the visual straight ahead task, only a few studies reported a slight leftward deviation of SSA in healthy right-handed subjects (Sumitani et al., 2007; Reinersmann et al., 2012), while others reported the overlap of SSA with the body mid-sagittal plane (Karnath et al., 1994, 2002; Vallar et al., 1999; Richard et al., 2004a,b; Saj et al., 2006, 2013; Rousseaux et al., 2013). The controversies among previous studies confounded the understanding of the processing of egocentric judgment in the brain.

Another critical issue is that the eye position could influence the judgment of SSA in normal subjects, which might cause the results of previous studies being not consistent. It was reported that the SSA biased either toward (Morgan, 1978) or opposite (Jeannerod and Biguer, 1989; Richard et al., 2005) to the fixation direction. Thus the controversies among previous SSA studies might be due to the influence of the eye position that was not properly monitored. Moreover, previous studies mainly focused on assessing the accuracy of egocentric discrimination, but the timing of egocentric judgment was unexplored.

To address these questions, in the present study, we measured the location of visual SSA and manual reaction time (RT) of healthy human subjects. Our egocentric discrimination task required subjects to keep fixation and press a key as quickly as possible to respond to the egocentric location of a visual cue. We found that the SSA of all subjects deviated to the left side of the body mid-sagittal plane. In addition, all subjects but one showed the longest RT at the location closest to the SSA; and in population, the RTs in the left visual field (VF) were longer than that in the right VF. Thus, our SSA and RT data demonstrate that the right hemisphere of healthy human subjects plays a more dominant role in processing egocentric information. Such results are consistent with the fact that hemispatial neglect more frequently occurs in patients with right hemisphere damage.

## Materials and methods

### Subjects

Sixteen healthy human subjects (age 22–29 years old, 7 males, 9 females) participated in this study. They were all right-handed subjects with normal or corrected to normal vision. We measured the eye dominance of each subject by 3 repeats of the “Hole-in-card” test (Miles, 1930), and the results were consistent for each individual subject. Among the 16 subjects, 5 subjects were left-eye dominant and 11 subjects were right-eye dominant. All subjects were naive to the experimental purpose. At the early stage of this study, 10 subjects (left-eye dominant: 5, right-eye dominant: 5) were recruited in the First Clinical College of Harbin Medical University. Since one subject had difficulty to keep fixation (the fixation break rate > 20%), data from this subject were excluded from further analysis. At the late stage of this study, the other 6 subjects (right-eye dominant: 6) were recruited in Beijing Normal University. The protocol of this study followed the ethical guidelines of the Declaration of Helsinki and was approved by the Institutional Review Board of the First Clinical College of Harbin Medical University and Beijing Normal University. All subjects gave written informed consent before participating in this study and received financial compensation for their participation.

### Apparatus

To eliminate the possibility that the surrounding objects might serve as the allocentric referees, all experiments were conducted in a dark room. The computer screen was placed 57 cm in front of the subjects' eyes. A chin rest restricted any head movement of subjects. When setting up the experimental system, we carefully measured the distance between the chin rest and the screen to ensure that: the chin rest and the screen were parallel in both horizontal and vertical dimensions, and the vertical middle line of the chin rest was aligned with the vertical middle line of the screen. Thus, we did our best to ensure that the screen was centered and oriented parallel to the plane of the chin rest. In addition, we marked the midpoint of the chin rest and instructed the subjects to put their chins on this point before collecting data in each session. And the subjects were instructed to keep the same posture throughout each session.

At the early stage of this study, a computer keyboard was positioned in front of the subjects, with the up and down keys aligned with their body mid-sagittal plane. The left key and right key were positioned with equal distance from the body mid-sagittal plane. At the late stage of this study, to exclude the possibility that the leftward deviation of keyboard might cause the deviation of SSA, the keyboard was covered by two black boxes with the same size, only leaving the up, down, left and right keys uncovered. In addition, the up and down keys were aligned with the body mid-sagittal plane.

All visual stimuli were presented on a 21-inch CRT monitor (SUN X7149A, 1280 × 960 pixels, 100 Hz vertical refresh rate) and the luminance of the stimuli was measured with a photometer (LS-110; Konica Minolta). Before starting the experiments, we calibrated and linearized the screen with the photometer. We also presented a circle with a 15° radius that was centered at the center of the screen. When we measured the distances from multiple points on the circle to the center of the screen, we found that the distances were all 15°. Thus, there was no spatial distortion on the screen display.

We monitored and collected the eye position signal at a sample rate of 1 kHz by an infrared camera eye tracking system (EyeLink 1000 Desktop Mount; SR Research). We used MATLAB (version 2012a; The MathWorks) with Psychtoolbox (PTB-3; Brainard and Pelli, 2015) to control the presentation of the visual stimuli and collect the subjects' RT data.

### Behavioral tasks

#### Egocentric discrimination task

**Figure 1:**
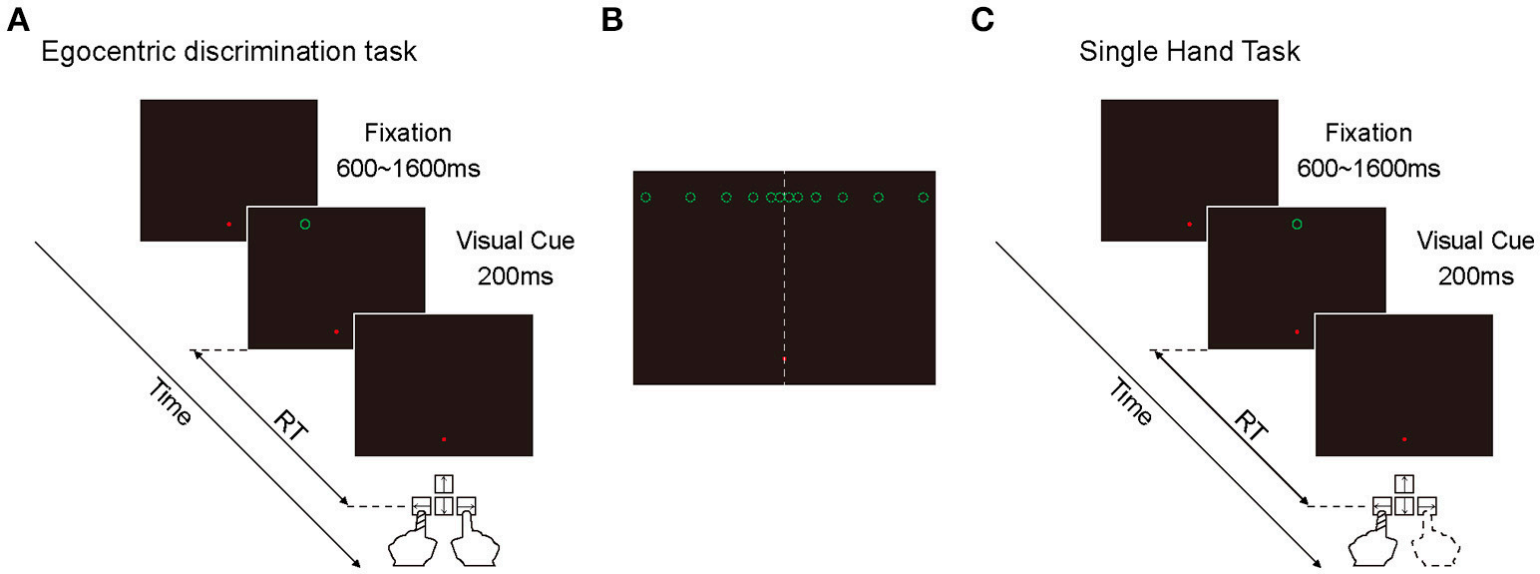
Illustration of the two behavioral tasks. **(A)** The diagram of egocentric discrimination task. **(B)** The illustration of the possible egocentric locations of the visual cue. **(C)** The diagram of single hand task.

Egocentric discrimination task (Main task, Figure 1A). The trial began with a red fixation point appearing on the screen with black background, located 9° below the center of the screen. The subjects needed to look at this fixation point within 500 ms and keep fixation within an invisible circular window (radius: 3°) until the end of the trial. If the eye position moved out of the fixation window during the fixation period, the fixation point would turn green and the trial would be terminated. After a random period of 600–1600 ms, a green circle (visual cue) would appear on the screen (radius: 0.5°, luminance: 0.05 cd/m^2^) for 200 ms. Subjects needed to judge whether the green circle was in the left or right side of the subjective mid-sagittal plane and press the left or right key accordingly (using the index of left or right hand, respectively) as quickly as possible.

At the early stage of this study, there were 12 possible locations in which the green circle randomly appeared at one of them. All 12 positions were 9° above the horizontal meridian of the screen in the Y-axis, and with 0.5, 1.5, 3.5, 6.5, 10.5, and 15.5° to the left and right in the X-axis (Figure 1B). During the experiments, the subjects performed 3 sessions per day for 3 days. Each session contained 120 trials. At the late stage of this study, there were only 6 possible locations (left and right 0.5, 1.5, and 3.5°) because the objective of the late stage was to assess the influence of eye position on spatial judgment. The inconsistency of the judgment to the same visual cue was high only at the center locations. During the experiments, the subjects performed 3 sessions per day for 2 days. Each session contained 120 trials.

Considering the possibility that the fixation point might serve as an allocentric cue to facilitate the egocentric judgment of the visual cue, we put the fixation point on the vertical meridian with 9° below the horizontal meridian, and put the visual cue with 9° above the horizontal meridian. Thus, when the visual cue appeared close to the vertical meridian, it was difficult for the subjects to judge the egocentric location of the visual cue based on its position relative to the fixation point.

#### Single hand task

Single hand task (Control task, Figure 1C). The sequence and temporal feature of this task were the same as the main task, with the only difference being that the green circle appeared only at one location: 9° above the center of the screen. In a given session, subjects were instructed to always press the same key (either left or right) as soon as the green circle appeared. In this task, subjects didn't need to make a judgment about the egocentric location of the visual cue. During the experiments, subjects performed 2 sessions daily (1 session with left key press and 1 session with right key press) at the beginning and the end of the experiment, respectively. Each session was composed of 30 trials. The sequence of left key press and right key press was randomized. This task was only performed at the early stage of this study.

### Data analysis

In the main task, we excluded fixation break trials (early stage: 4.60%, 447 out of 9,720 trials; late stage: 4.35%, 188 out of 4,320 trials) and no response trials (early stage: 0.01%, 1 out of 9,720 trials; late stage: 0.14%, 6 out of 4,320 trials) from data analysis. In the control task, we first excluded fixation break trials (4.42%, 138 out of 3,120 trials). Then trials with RT differing more than 3 standard deviations from the mean RT of each day (in total, 1.48%, 44 out of 2,982 trials) were also excluded. The number of no response trials was zero.

#### Point of subject equality (PSE) calculation

For each subject, the manual response (e.g., the percentage of pressing right key) at each visual cue location was calculated. A cumulative normal distribution function was used to fit the response percentage data to estimate the psychometric function. And then we defined the point of subject equality (PSE) of each psychometric curve as the point (location in horizontal dimension) at which the percentage of leftward and rightward response were equal (50%). The PSE was regarded as the location of the visual SSA of every subject. Negative value indicated a leftward deviation whereas positive value indicated a rightward deviation from the body mid-sagittal plane.

#### RT calculation

We intended to explore the egocentric judgment time by measuring the manual RT of two hands. However, considering the RTs between two hands often differ in humans (intrinsic RT difference) (Boulinguez et al., 2001), we should first exclude the influence of the intrinsic RT difference. Thus we calculated the intrinsic RT difference in the control task (single hand task, Figure 1C) by subtracting the mean RTs of the right hand from the mean RTs of the left hand. Then we calculated the post-adjusted RTs in the main task by subtracting the intrinsic RT difference from the RTs of the left hand. The RT data presented in this paper were post-adjusted. We also excluded outliers in which the post-adjusted RT differed more than 3 standard deviations from the mean post-adjusted RT of each day (in total, 1.24%, 115 out of 9,272 trials).

## Results

At the early stage of this study, 9 subjects performed both the main task (12 visual cue locations) and the control task. The RTs of each individual subject in the control task were relatively consistent across the experimental days, as is shown in Table 1. The results of each individual subject's judgment at each visual cue location in the main task are presented in Table 2. Unsurprisingly, the inconsistent judgment (same visual cue location ended with opposite judgments) occurred more frequently when the visual cue was close to the body mid-sagittal plane. Notably, the frequency of inconsistent judgment was not symmetric between the two most center locations (−0.5° versus 0.5°), but with significantly higher rate at −0.5° (*p* = 4.1135e-05, Wilcoxon test). The greater rate of inconsistent judgment at −0.5° indicated that, compared with 0.5°, it was more difficult to judge the egocentric location of the visual cue at −0.5°.

**Table 1:** Mean RTs of the left and right hand and the intrinsic RT difference between the two hands (left hand–right hand) of individual subject in the control task (unlike the other subjects, subject WG performed the main task with 180 trials per session and completed the experiments within 2 days).

**Subject**	**Mean RTs of left hand (ms)**				**Mean RTs of right hand (ms)**				**Intrinsic RT difference between two hands (ms)**			
	**Day 1**	**Day 2**	**Day 3**	**Mean**	**Day 1**	**Day 2**	**Day 3**	**Mean**	**Day 1**	**Day 2**	**Day 3**	**Mean**
CJL	425.85	397.27	364.77	395.96	457.08	400.37	378.85	412.10	−31.22	−3.11	−14.08	−16.14
DYC	410.44	397.13	342.86	383.48	379.89	354.50	328.90	354.43	30.55	42.63	13.96	29.05
GZ	318.40	305.40	292.78	305.53	315.93	301.27	291.05	302.75	2.47	4.13	1.73	2.78
LMJ	310.28	307.05	293.91	303.75	294.89	284.74	293.85	291.16	15.39	22.31	0.05	12.58
LYH	481.99	437.30	413.16	444.15	441.94	433.73	443.68	439.79	40.05	3.56	−30.52	4.37
QHL	348.12	331.61	305.50	328.41	358.75	319.85	303.08	327.22	−10.63	11.76	2.43	1.18
QSH	362.37	354.65	349.29	355.44	363.84	381.07	344.39	363.10	−1.47	−26.41	4.90	−7.66
WG	362.71	338.14	–	350.43	373.65	349.32	–	361.48	−10.94	−11.17	–	−11.06
ZHH	333.26	326.13	320.14	326.51	309.98	306.88	311.01	309.29	23.28	19.25	9.13	17.22

**Table 2:** The egocentric judgments of individual subject in the main task.

**Subject**	**Location of visual cue**											
	**Left VF**						**Right VF**					
	**−15.5°**	**−10.5°**	**−6.5°**	**−3.5°**	**−1.5°**	**−0.5°**	**0.5°**	**1.5°**	**3.5°**	**6.5°**	**10.5°**	**15.5°**
**EARLY STAGE**												
CJL	80/0	86/0	81/0	81/0	74/**4**	37/**44**	79/**6**	82/**2**	80/0	83/0	82/0	86/0
DYC	87/0	85/0	85/0	84/0	81/**5**	50/**31**	68/**14**	84/**2**	83/0	88/0	83/0	87/0
GZ	89/0	88/0	89/0	86/**1**	81/**6**	54/**34**	72/**16**	87/0	86/**1**	89/0	88/0	88/0
LMJ	79/0	95/0	92/0	92/0	82/**11**	51/**37**	71/**20**	87/**1**	91/**1**	79/0	79/0	78/0
LYH	88/0	85/0	84/0	88/0	80/**6**	55/**33**	81/**7**	85/**1**	87/**1**	84/0	82/0	83/0
QHL	86/0	81/0	86/0	88/0	73/**11**	37/**48**	68/**16**	88/**2**	88/0	87/0	86/0	87/0
QSH	89/0	89/0	88/0	87/**1**	89/**1**	57/**31**	66/**19**	84/**4**	90/0	90/0	89/0	88/0
WG	84/0	88/0	88/0	90/0	88/**1**	54/**26**	86/**3**	88/0	85/0	84/0	86/0	83/0
ZHH	83/0	84/0	84/0	83/0	81/**1**	63/**22**	70/**15**	83/0	84/0	86/0	83/0	87/0
Total	765/0	781/0	777/0	779/**2**	729/**46**	458/**306**	661/**116**	768/**12**	774/**3**	770/0	758/0	767/0
**LATE STAGE**												
DY	-	-	-	117/**2**	120/0	84/**36**	111/**9**	118/**2**	118/0	-	-	-
JFF	-	-	-	113/0	108/**4**	87/**26**	100/**11**	107/0	118/0	-	-	-
LM	-	-	-	110/0	110/**2**	73/**33**	105/**6**	116/0	109/0	-	-	-
XF	-	-	-	109/**2**	110/0	87/**27**	95/**20**	114/**1**	112/0	-	-	-
YL	-	-	-	116/0	114/**2**	71/**45**	104/**13**	119/0	117/**1**	-	-	-
ZZY	-	-	-	117/0	117/0	95/**23**	98/**11**	116/**3**	111/0	-	-	-
Total	-	-	-	682/**4**	679/**8**	497/**190**	613/**70**	690/**6**	685/**1**	-	-	-

*This table shows the number of trials, with leftward/rightward judgment in the left VF and rightward/leftward judgment in the right VF. The numbers of trials with rightward judgment in the left VF and leftward judgment in the right VF are highlighted in bold*.

### The PSE of the psychometric curve of each subject deviated to the left side of the body mid-sagittal plane

**Figure 2:**
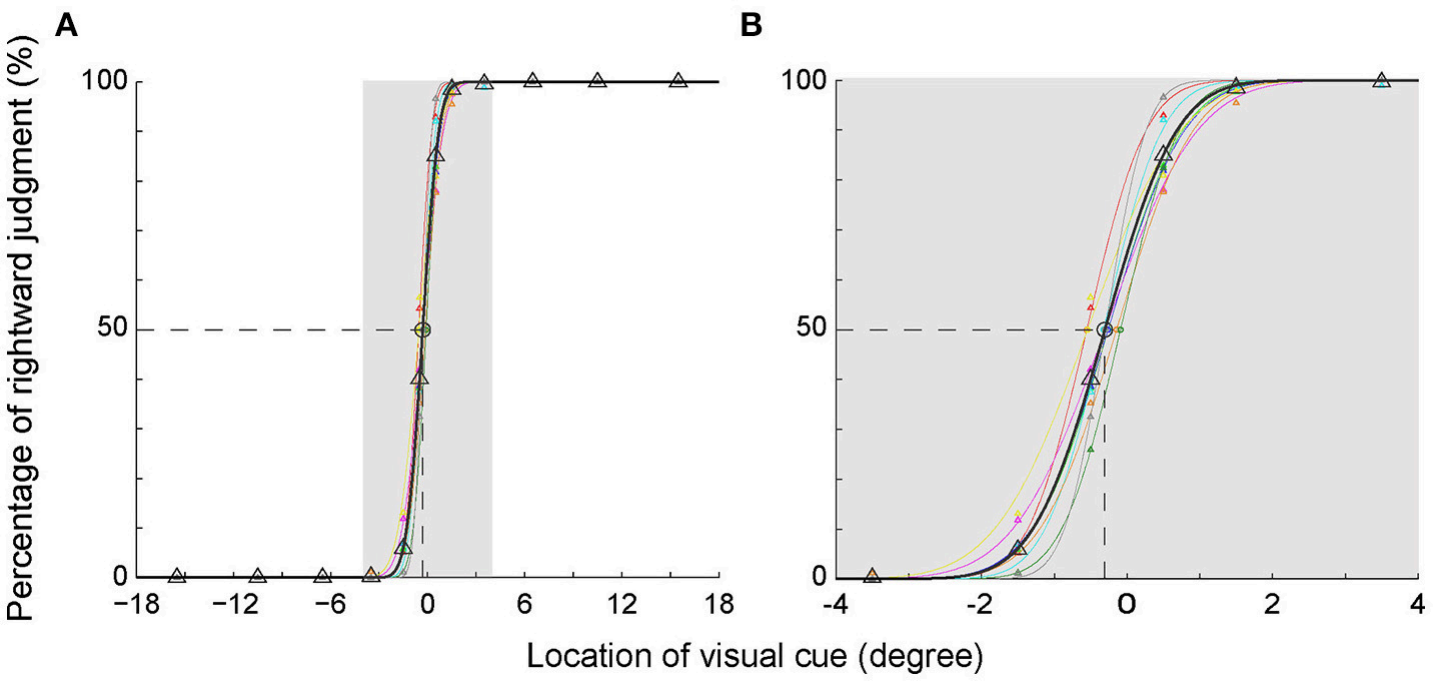
Percentage of the rightward judgment as a function of the egocentric location of the visual cue. **(A)** The psychometric curve of each individual subject (different colors) and the averaged psychometric curve of population data (black). **(B)** The enlarged graph of gray area in panel A for clearer vision. The PSEs of all subjects shifted to the left side of the body mid-sagittal plane, with a population PSE: −0.31° ± 0.16° (mean ± SD).

To illustrate the subjects' egocentric judgment more clearly, we analyzed the percentage of rightward judgment at each visual cue location. To be noticed, the percentage of leftward judgment was a mirror image of the percentage of rightward judgment (100–the percentage of rightward judgment). The percentages of rightward judgment at each visual cue location in the main task are shown in Figure 2A. Data of each individual subject (same colored triangles) were fitted with a cumulative normal distribution function, shown as the same colored thin curve. The averaged data were denoted as black triangles and thick curve. As expected, the percentages of rightward judgment at the 3 most peripheral locations in the left VF (−15.5, −10.5, and −6.5°) were all 0% and those at the 3 most peripheral locations in the right VF (15.5, 10.5, and 6.5°) were all 100% among the subjects. When the visual cue got closer to the body mid-sagittal plane (from −3.5 to 3.5°), the percentage of rightward judgment gradually increased in the left VF, and it gradually decreased in the right VF (Figure 2B). Such results indicated that, when the visual cue was close to the body mid-sagittal plane, the egocentric judgment to the same location was ended with opposite outputs (leftward versus rightward, Table 2). Strikingly, the fitted psychometric curves of all subjects shifted to the left side of the body mid-sagittal plane, and the population point of subject equality (PSE) was at −0.31° ± 0.16° (mean ± SD). Compared with the body mid-sagittal plane, the leftward deviation of SSA was significant (*p* = 4.1135e-05, Wilcoxon test). These results showed that the visual SSA of all subjects deviated to the left side of the body mid-sagittal plane, regardless of the eye dominance.

### The RTs were longer when the visual cue was closer to the SSA rather than the body mid-sagittal plane

**Figure 3:**
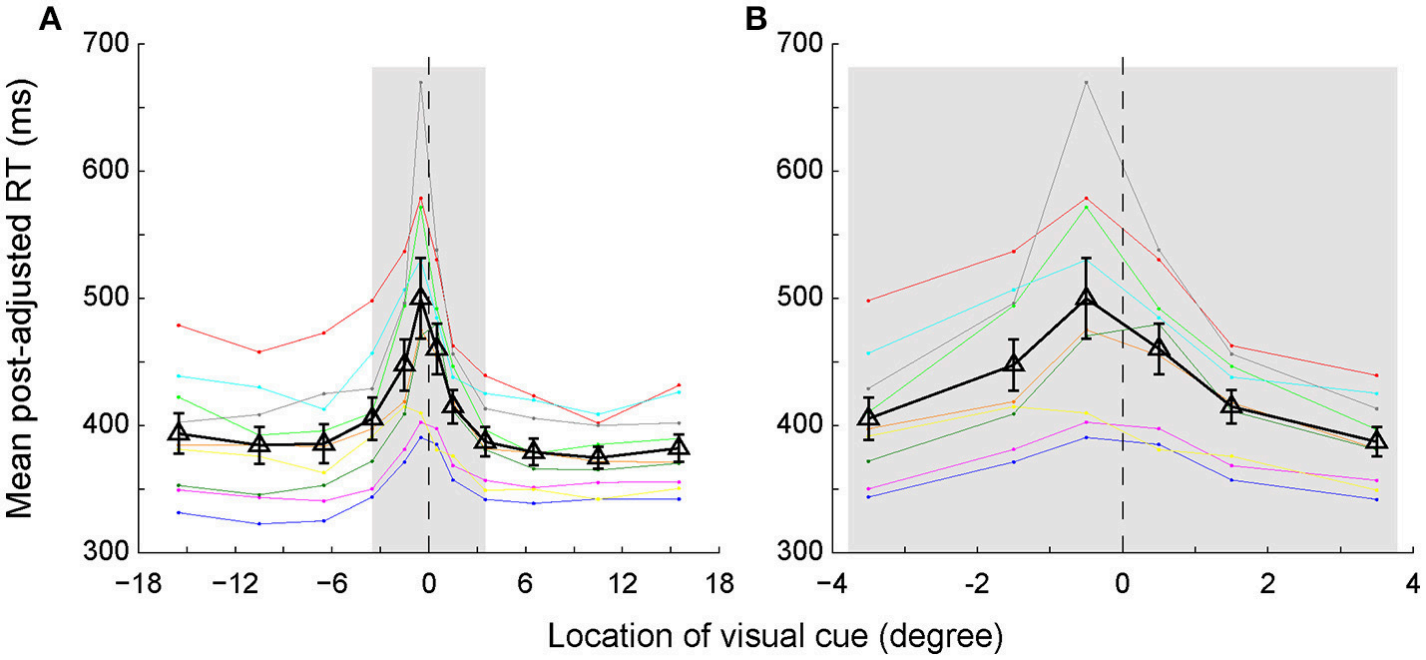
The post-adjusted RTs distribution as a function of the egocentric location of the visual cue. **(A)** Same colored dots connected with the same colored lines represent one individual subject's RTs. Black triangles connected with black lines represent the averaged RTs of all subjects. The vertical bars represent the standard errors. **(B)** The enlarged graph of gray area in panel A for clearer vision.

We assessed the time of egocentric judgment by calculating the post-adjusted RTs of each individual subject in the main task (Figure 3A). As expected, the RTs gradually prolonged as the visual cue got closer to the body mid-sagittal plane. Notably, the RTs were asymmetric between left and right VFs: RTs were longer in the left VF than in the right VF. Two-way ANOVA showed that both eccentricity [*F*_(5, 96)_ = 10.92, *p* = 2.4673e-08, η_p_^2^ = 0.36] and laterality [*F*_(1, 96)_ = 4.12, *p* = 0.045, η_p_^2^ = 0.04] of the visual cue had significant influence on the RTs. But there was no significant interaction effect between the two factors [*F*_(5, 96)_ = 0.32, *p* = 0.901, η_p_^2^ = 0.02]. Moreover, RTs of all subjects but one were longest when the visual cue was closest to the SSA. For instance, the population RT was longest when the visual cue was at −0.5° (Figure 3B), consistent with the fact that the mean SSA was located at −0.31° (Figure 2B).

**Figure 4:**
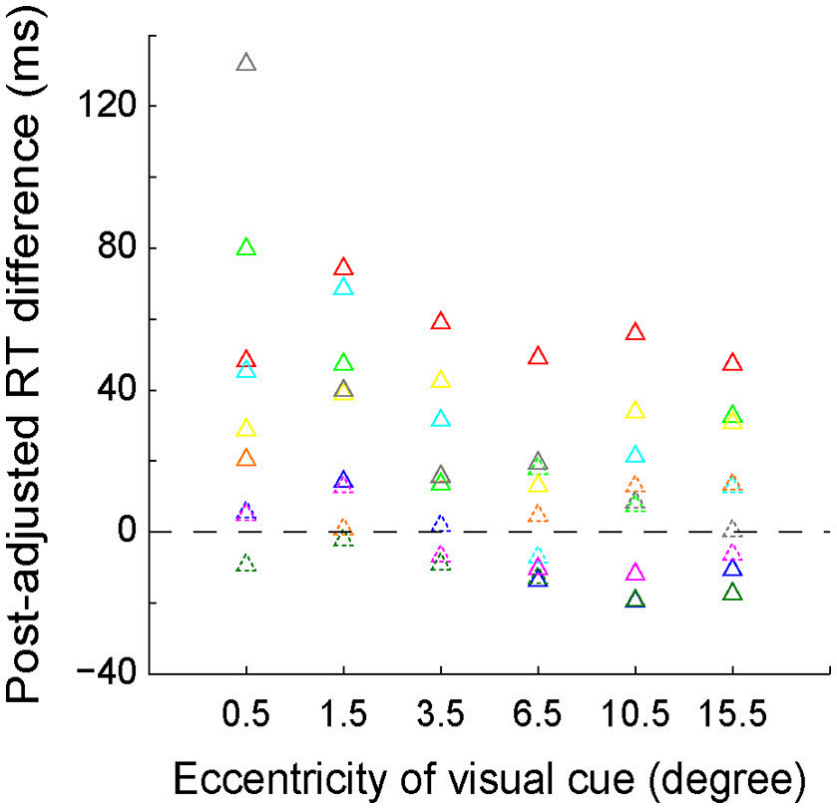
The distribution of post-adjusted RT difference (left VF–right VF) between the 6 mirror locations of the visual cue. The same colored triangles represent the data of one individual subject. Solid symbols indicate that the RT difference reached the statistically significant level (*p* < 0.05, Wilcoxon test), whereas the dashed symbols do not.

We further quantitatively analyzed the RT difference (left VF–right VF) between each mirror location for each individual subject. The results of the subtracted RTs of each subject are presented with different colored triangles in Figure 4. The solid symbols denote that the RT difference between left and right VFs reached the statistically significant level (*p* < 0.05, Wilcoxon test), whereas the dashed symbols do not. As shown, the RT difference between the 2 most center locations (−0.5° versus 0.5°) was greatest, with the RT difference of 6 out of 9 subjects reaching the statistically significant level. The RT difference gradually decreased as the eccentricity of visual cue increased. Eventually, at the peripheral locations, the RT difference of several subjects reversed.

The longer RT indicated that longer time was needed to judge the egocentric location of the visual cue. Our RT data showed that the most difficult egocentric judgment was around the SSA, rather than the body mid-sagittal plane.

### The leftward deviation of SSA was not due to the influence of eye position

At the late stage of this study, 6 subjects performed the main task with only 6 visual cue locations. The objective of the late stage was to assess the influence of eye position on the spatial judgment to a visual cue. We compared the eye position (averaged from visual cue onset to 250 ms later) between trials where subjects made two opposite judgments to the same visual cue.

**Figure 5:**
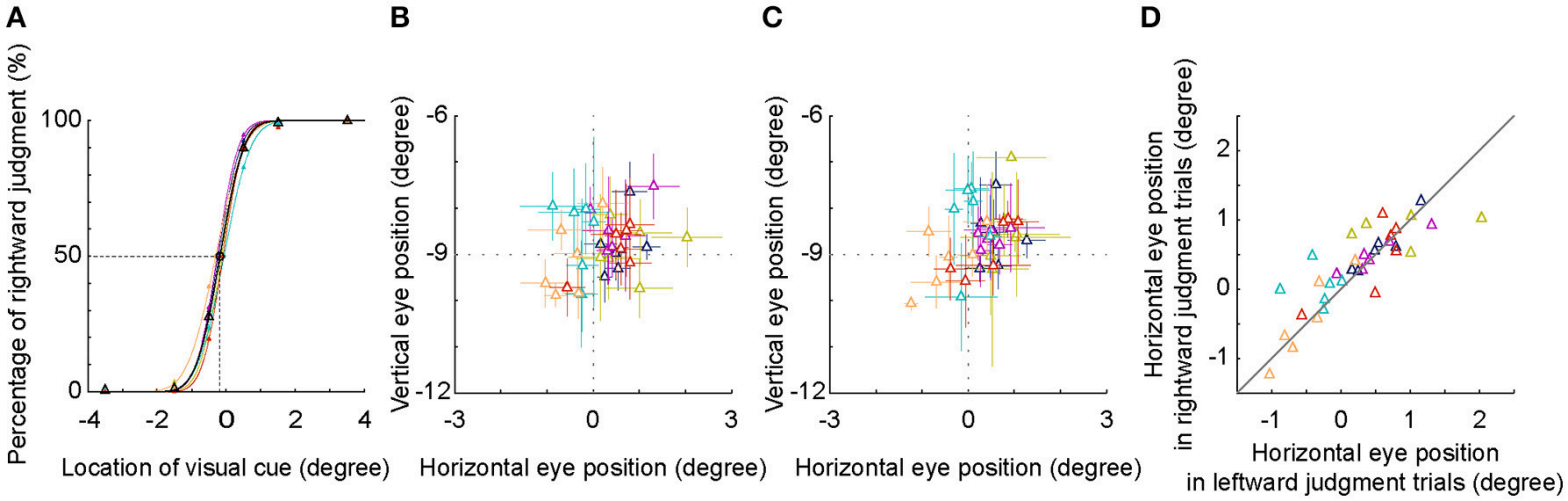
SSA and eye position analysis. **(A)** The psychometric curve of each individual subject (different colors) and the averaged psychometric curve of population data (black). The PSEs of all subjects shifted to the left side of the body mid-sagittal plane, with a population PSE: −0.18° ± 0.10° (mean ± SD). **(B,C)** Averaged eye position during an interval between visual cue onset and 250 ms later. Visual cue was judged as in the left side **(B)** or as in the right side **(C)**. **(D)** Comparison of horizontal eye positions between trials with leftward and rightward judgments. Each symbol represents the data of one session. Same colored symbols represent the data of one individual subject.

First, the fitted psychometric curves of the rightward judgment percentages of 6 subjects shifted to the left side of the body mid-sagittal plane. The population PSE was at −0.18° ± 0.10° (mean ± SD) (Figure 5A). Compared with the body mid-sagittal plane, the leftward deviation of SSA was significant (*p* = 0.002, Wilcoxon test), which was consistent with the results of 9 subjects at the early stage.

The location of −0.5° was nearest to the location of SSA, thus the inconsistency of judgment (leftward versus rightward) was highest. We compared the eye positions between two groups of trials at −0.5°. We found that the distribution of the eye position was centered at the fixation point, regardless of the results of spatial judgment (Figures 5B,C). Furthermore, the horizontal eye positions between the two groups of trials were not significantly different both in the individual subject level (except one subject) and in the population level (Figure 5D, *p* = 0.496, Wilcoxon test). These results indicated that in the present study, the leftward deviation of SSA was not due to the influence of eye position.

## Discussion

In the present study, we assessed the egocentric judgment of healthy subjects by measuring the location of SSA and manual RT. To exclude the influence of eye position on egocentric judgment, we asked the subjects to keep fixation and monitored the eye position by an infrared camera eye tracking system during the experiments. We found that: (1) the SSA of the subjects all deviated to the left side of the body mid-sagittal plane; (2) the RTs of all subjects but one were longest when the visual cue was nearest to the location of SSA, with the RTs in the left VF being longer than that in the right VF; (3) there was no significant difference of horizontal eye positions between the trials with opposite spatial judgments.

### The possible interpretation of our results

The anatomical and physiological features of visual system of primates might explain our results—the leftward deviation of SSA and longer RT in the left VF. Previous studies have revealed that, while the lower visual areas in one hemisphere strictly process the visual information from contralateral VF (Tootell et al., 1982), the higher visual cortices process the visual information from both contralateral and ipsilateral VFs (Gross et al., 1969, 1972; Andersen et al., 1990; Raiguel et al., 1997; Ben Hamed et al., 2001). One reasonable explanation for our results is that the right hemisphere receives and processes more visual information from the ipsilateral VF than the left hemisphere does (Sheremata et al., 2010; Zhou et al., 2012; Sheremata and Silver, 2015). Therefore, compared with the left VF, the right VF might be overestimated, so that the SSA deviated to the left VF and the sensorimotor processing took less time in the right VF, i.e., shorter RTs. A strongly supportive evidence for this assumption is that the serious hemispatial neglect more frequently occurs after lesions of the right hemisphere but not after lesions of the left hemisphere (Beis et al., 2004; Ringman et al., 2004; Becker and Karnath, 2007; Kleinman et al., 2007).

### SSA deviates to the left side when the eyes fixate straight ahead

It is well-known that the eye position strongly affects the perception of visual egocentric information (Barbeito and Simpson, 1991; Sridhar and Bedell, 2011, 2012). However, most previous studies did not monitor the eye position during experiments, thus the effect of eye position on visual egocentric judgment was ignored. The importance of eye position on visual egocentric judgment is caused by the fact that the retinal visual input combines with the eye position signal to build a head-centered reference frame. At the same time, the retinotopic position of an object is also transformed into the head-centered reference frame (Andersen et al., 1997). Indeed, the effect of eye position on the judgment of SSA in normal subjects has been found in previous studies (Morgan, 1978; Jeannerod and Biguer, 1989; Richard et al., 2005).

To exclude the effect of eye position on the judgment of SSA, we asked subjects to keep fixating straight ahead during the experiments and monitored the subjects' eye position. We found that the horizontal eye positions between the trials with opposite spatial judgments were not significantly different. Thus, the leftward deviation of SSA in the present study was not caused by the deviation of eye position.

### The importance of measuring the location of SSA in study of egocentric representation

The location of SSA is frequently measured in the studies of egocentric representation in healthy subjects and brain damage patients. SSA reflects the location of the subjective mid-sagittal plane, which subjectively separates the egocentric space into left and right halves. Thus, the comparison between the location of SSA and body mid-sagittal plane can provide useful information to help to understand the contribution of the two hemispheres to egocentric information processing. Based on the findings from previous studies, many factors can cause the deviation of SSA, which may share different neural mechanisms. First, the SSA will deviate toward the ipsilateral side of sensory stimulation if it is presented only in one side, including neck proprioception stimulation (Karnath et al., 1994, 2002), vestibular stimulation (Karnath et al., 1994) and acute experimental painful stimulation (Bouffard et al., 2013). The deviations of SSA under these conditions are mainly due to the post-training effect, which is very different from the mechanisms leading to the deviation of SSA in our present study. Second, patients with lateralized lesion of the peripheral nervous system or visual cortices also show the deviations of SSA, such as unilateral pathologic pain (Sumitani et al., 2007; Reinersmann et al., 2012), left vestibular loss (Saj et al., 2013) and homonymous hemianopia (Ferber and Karnath, 1999; Rousseaux et al., 2013) or quadrantanopia (Kuhn et al., 2010). Third, damage in cortical regions, in particular the parietal cortex, results in the symptom of hemispatial neglect and the ipsilesional deviation of SSA (Karnath, 1994; Farne et al., 1998; Ferber and Karnath, 1999; Schindler and Kerkhoff, 2004; Richard et al., 2004a,b, 2005; Saj et al., 2006; Rousseaux et al., 2013). These findings from clinical studies indicate that the unbalanced egocentric information processing between the two hemispheres causes the deviation of SSA. Here we report the leftward deviation of SSA in healthy human subjects when they fixate straight ahead, which might also reflect the asymmetric process of egocentric information between the two hemispheres.

## Conclusion

In the present study, we found that when the eyes fixated straight ahead, the visual SSA of healthy human subjects deviated to the left VF and the manual RT was longer in the left VF than in the right VF. Such results suggest that the egocentric information is asymmetrically processed between the two hemispheres.

## Author contributions

YZ, MZ, and YP designed the experiments; YZ and GW collected the data; YZ and BL analyzed the data; YZ prepared all figures; YZ, MZ, and YP wrote and revised the manuscript; MZ and YP supervised the experiments.

### Conflict of interest statement

The authors declare that the research was conducted in the absence of any commercial or financial relationships that could be construed as a potential conflict of interest.

## Abbreviations

PSE: point of subject equality
RT: reaction time
SSA: subjective straight ahead
VF: visual field.
